# Extracorporeal membrane oxygenation for respiratory failure in children: the years before and after the 2009 H1N1 pandemic

**DOI:** 10.5935/0103-507X.20210082

**Published:** 2021

**Authors:** Felipe Rezende Caino de Oliveira, Orlei Ribeiro de Araujo, Daniel Garros, José Colleti Junior, Werther Brunow de Carvalho, Laurance Lequier

**Affiliations:** 1 Pediatric Intensive Care Unit, Hospital Santa Catarina, São Paulo, São Paulo (SP), Brazil.; 2 Pediatric Intensive Care Unit, Grupo de Apoio ao Adolescente e à Criança com Câncer, Instituto de Oncologia Pediátrica, Universidade Federal de São Paulo - São Paulo (SP), Brazil.; 3 Pediatric Intensive Care Unit, Stollery Children’s Hospital - Edmonton, Alberta, Canada.

**Keywords:** Respiratory insufficiency, Extracorporeal membrane oxygenation, H1N1, Influenza A virus, H1N1 Subtype, Influenza, human, Respiratory distress syndrome, ARDS, Pandemics, Survival rate, Child

## Abstract

**Objective:**

To evaluate whether there was any impact on the number of pediatric extracorporeal membrane oxygenation runs and survival rates in the years subsequent to the 2009 pandemic.

**Methods:**

We studied two different periods of extracorporeal membrane oxygenation support for respiratory failure in children by analyzing datasets from the Extracorporeal Life Support Organization. Autoregressive integrated moving average models were constructed to estimate the effect of the pandemic. The year 2009 was the year of intervention (the H1N1 epidemic) in an interrupted time series model. Data collected from 2001 - 2010 were considered preintervention, and data collected from 2010 - 2017 were considered postintervention.

**Results:**

There was an increase in survival rates in the period 2010 - 2017 compared to 2001 - 2010 (p < 0.0001), with a significant improvement in survival when extracorporeal membrane oxygenation was performed for acute respiratory failure due to viral pneumonia. The autoregressive integrated moving average model shows an increase of 23 extracorporeal membrane oxygenation runs per year, prior to the point of the level effect (2009). In terms of survival, the preslope shows that there was no significant increase in survival rates before 2009 (p = 0.41), but the level effect was nearly significant after two years (p = 0.05), with a 6% increase in survival. In four years, there was an 8% (p = 0.03) increase in survival, and six years after 2009, there was up to a 10% (p = 0.026) increase in survival.

**Conclusion:**

In the years following 2009, there was a significant, global incremental increase in the extracorporeal membrane oxygenation survival rates for all runs, mainly due to improvements in the technology and treatment protocols for acute respiratory failure related to viral pneumonia and other respiratory conditions.

## INTRODUCTION

Respiratory failure is a frequent cause of admission to the pediatric intensive care unit (PICU). In children, the mortality rates for respiratory failure related to viral or bacterial pneumonia, trauma, and acute respiratory distress syndrome (ARDS) are still unacceptably high. Severe viral lower respiratory tract infections, including influenza, can progress to acute respiratory distress syndrome by means of host and viral mechanisms, which include epithelial cell death, alveolar compromise, local and systemic cytokine production, innate immune cellular infiltration, exuberant T cell responses, and other innate and adaptative immune responses.^(^1^)^ Acute respiratory distress syndrome is routinely managed using lung protective ventilator strategies, but if these ventilation strategies cannot provide adequate oxygenation, patients may require extracorporeal membrane oxygenation (ECMO), which has increasingly been gaining importance as a salvage therapy.^(^2^,^3^)^ The main indications for ECMO are acute severe heart or lung failure with high risk for mortality despite optimal conventional therapy. Thus, ECMO is considered when a 50% mortality risk is predicted.^(^4^)^

In recent decades, health systems worldwide have been confronting new epidemic and pandemic infections. During the 2009 H1N1 pandemic, hundreds of patients with ARDS worldwide received ECMO.^(^5^)^ The proportion of ECMO use for influenza-associated disease has increased over time, with a peak in 2009. The overall survival rate (all ages, all centers around the world) for ECMO during the 2009 pandemic was 60%, as reported by de St Maurice et al. in a study that explored the International Extracorporeal Life Support Organization (ELSO) database.^(^6^)^ The ELSO is an organization intended to assist institutions in delivering extracorporeal life support through education, guideline development, original research, publications, and maintenance of a comprehensive registry that, in 2020, included data on more than 130,000 patients.^(^7^)^

As the applications of ECMO in children grow, the analysis of outcomes is becoming increasingly important to ensure that this therapy remains available for appropriate candidates and to ensure better long-term survival and functional prognosis.^(^8^)^

This study evaluated, in a historical series, whether in the years subsequent to the 2009 pandemic there has been any impact on the number of pediatric ECMO runs and survival rates.

## METHODS

We analyzed the summary datasets from the ECMO Registry of Extracorporeal Life Support Organization (ELSO, Ann Arbor, MI, https://www.elso.org/)

Data from patients aged 1 month to 18 years were included and used to calculate the ECMO usage and survival (to hospital discharge) rates. The frequencies were analyzed using chi-square and Fisher’s exact tests, with 95% confidence intervals (95%CIs) and a significance level of 0.05. We built a time series using the data available for the total number of pediatric respiratory runs with the interrupted time series method, whose characteristics are the data collected at multiple points before and after an intervention.^(^9^)^ We used 2009 as the year of “intervention” (the H1N1 pandemic). Data collected from 2001 - 2010 were considered “preintervention”; 2009 data were received by ELSO and compiled until July 2010 (the “intervention” year); and data collected from July 2010 - 2017 were considered “postintervention”. Autoregressive integrated moving average (ARIMA) models were constructed, and trends and autocorrelation were considered to estimate the effect of the pandemic using Statistical Package for the Social Sciences (SPSS), version 20.0 (IBM Corp. Armonk, NY).

## RESULTS

**Figure 1:**
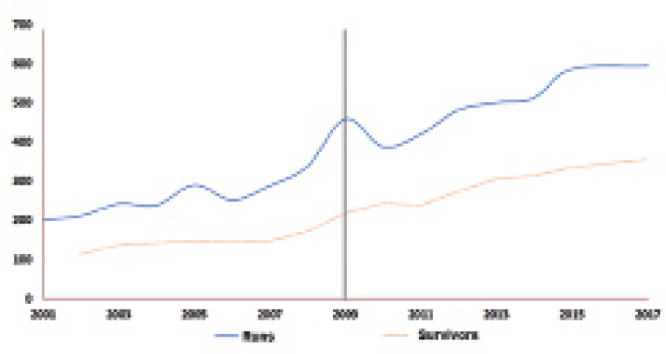
Time series of the number of respiratory runs and number of survivors.

The ECMO runs in the preintervention and postintervention periods are displayed in table 1, where we can observe increased survival rates for all runs in the second period (2010 - 2017) compared with the period (between 2001 and 2010) (p < 0.0001). Table 1 also shows the ECMO runs by diseases and conditions. We observed a significant improvement in the survival rates when ECMO was performed for acute respiratory failure due to viral pneumonia and in other respiratory conditions. However, there was no improvement in survival for other forms of acute respiratory failure secondary to lung disease (non-ARDS diagnosis, aspiration pneumonia and bacterial pneumonia), ARDS in patients who required surgery after trauma, and ARDS unrelated to surgery. In the ARIMA model (Table 2), the preslope coefficient tells us that there was an increase of 23 ECMO runs per year, prior to the point of the level effect (2009), and no effect level after this point. In terms of survival, the preslope shows that there was no significant increase before 2009 (p = 0.41), but the level effect was nearly significant within two years (p = 0.05), with a 6% increase in survival. In four years, there was an 8% increase in the survival rate (p = 0.03), and the survival rate increased to 10% six years after 2009 (p = 0.026). The time series of the number of respiratory runs and number of survivors is illustrated in figure 1.

## DISCUSSION

This study compared two distinct periods of use of ECMO support for respiratory failure: the years before and the years after the 2009 H1N1 pandemic. The increase in the number of pediatric respiratory runs, following a trend since the beginning of the 2000s, was unrelated to the pandemic, according to the ARIMA model. This model, however, suggests that the events that occurred in 2009 influenced the improvement in survival rates. The overall survival rate increased 6% in the two subsequent years, after having remained relatively unchanged for several decades.^(^10^)^ When ECMO was used as a rescue therapy for respiratory failure secondary to viral pneumonia, the survival rates improved significantly, from 65.8% to 72.5%. Furthermore, improvements in survival rates were also observed for veno-venous ECMO, increasing from 65.4% to 69.8%. These increments can be attributed, at least partially, to advances in the technology, which included refinement of the double lumen veno-venous cannulas to support a large range of patient sizes with less recirculation, pumps with lower prime volumes, more efficient oxygenators, and changes made in the circuit configuration to decrease turbulent flow and hemolysis.^(^11^)^ Unfortunately, during the influenza pandemic, no randomized clinical trial for patients with H1N1 was proposed or completed due to logistical and ethical reasons.^(^12^)^ In a systematic review and meta-analysis that included 8 studies and 266 patients with acute lung injury due to H1N1 influenza infection who received ECMO support, Zangrillo et al. maintained that ECMO implementation can be recommended in selected centers, since training, logistics and resources are adequate. In this meta-analysis, outcomes were highly variable among the studies, with in-hospital or short-term mortality rates ranging from 8% to 65%. None of these studies included children.^(^13^)^ To date, no clinical trials have established the efficacy of ECMO for pediatric respiratory failure.^(^14^)^ It is important to recognize, however, that veno-venous support carries a lower risk of central nervous system injury and mortality, and this mode should be the default choice for pediatric respiratory failure.^(^15^)^ However, the size of the double cannulas and their widespread availability remain a challenge.^(^10^)^ With improvements in both oxygenators and pump technology, the management of patients on ECMO has become simpler, but a successful ECMO run requires accurate and safe placement of a suitably sized cannula.^(^16^)^

**Table 1:** Estracorporeal membrane oxygenation in children in two periods: before and after 2009 (2009 data are compiled until July 2010)

	Runs (n)	Deaths n (%)	Survivors n (%)	Relative risk (95%CI)	Odds ratio (95%CI)	p value
All runs						
July 2001 - July 2010	2,490	1,096 (44.01)	1,394(55.9)	1.14 (1.07 - 1.21)	1.25 (1.12 - 1.39)	< 0.0001
July 2010 - July 2017	3,290	1,268 (38.5)	2,022 (61.5)			
Viral pneumonia						
July 2001 - July 2010	365	125 (34.2)	240 (65.8)	1.24 (1.02 - 1.51)	1.37 (1.03 - 1.82)	0.037
July 2010 - July 2017	541	149 (27.5)	392 (72.5)			
Bacterial pneumonia						
July 2001 - July 2010	303	120 (39.6)	183 (60.4)	1.16 (0.93 - 1.46)	1.27 (0.89 - 1.82)	0.2
July 2010 - July 2017	239	81 (33.8)	158 (66.1)			
Aspiration pneumonia						
July 2001 - July 2010	42	12 (28.6)	30 (71.4)	0.86 (0.48 - 1.5)	0.81 (0.35 - 1.88)	0.67
July 2010 - July 2017	70	23 (32.8)	67.2)			
ARDS in postoperative of trauma						
July 2001 - July 2010	63	25 (39.7)	38 (60.3)	1.09 (0.63 - 1.88)	1.15 (0.48 - 2.75)	0.9
July 2010 - July 2017	33	12 (36.4)	21 (63.6)			
ARDS nonpostoperative						
July 2001 - July 2010	173	79 (45.7)	94 (54.3)	1.11 (0.87 - 1.41)	1.2 (0.78 - 1.84)	0.47
July 2010 - July 2017	165	68 (41.2)	97 (58.8)			
Acute respiratory failure, non-ARDS						
July 2001 - July 2010	242	102 (42.1)	140 (57.9)	1.13 (0.94 - 1.35)	1.22 (0.9 - 1.66)	0.23
July 2010 - July 2017	562	210 (37.4)	352 (62.6)			
Other respiratory runs						
July 2001 - July 2010	1,336	655 (49)	681 (51)	1.12 (1.04 - 1.21)	1.24 (1.07 - 1.43)	0.004
July 2010 - July 2017	1,632	714 (43.8)	918 (56.3)			
Veno-arterial runs						
July 2001 - July 2010	1,180	588 (49.8)	592 (50.2)	1.07 (0.99 - 1.16)	1.14 (0.98 - 1.33)	0.1
July 2010 - July 2017	1,434	668 (46.6)	766 (53.4)			
Veno-venous runs						
July 2001 - July 2010	853	295 (34.6)	558 (65.4)	1.15 (1.02 - 1.29)	1.22 (1.03 - 1.45)	0.023
July 2010 - July 2017	1,897	572 (30.2)	1,325 (69.8)			

95%CI - 95% confidence interval; ARDS - acute respiratory distress syndrome. The odds ratios refer to the probabilities of survival, comparing the runs in the two periods.

**Table 2:** Autoregressive integrated moving average (ARIMA) model

	Estimate coefficient	Standard error	p value
Pediatric respiratory runs			
Preslope	23.2	10.6	0.049
Level effect (2 years)	49.2	82.3	0.5
Level effect (4 years)	9.2	96.6	0.9
Level effect (6 years)	-30.8	121.9	0.8
Survival			
Preslope	-0.003	.004	0.4
Level effect (2 years)	0.06	.030	0.05
Level effect (4 years)	0.08	.034	0.03
Level effect (6 years)	0.1	.041	0.026

The upper line shows the temporal evolution of the number of respiratory runs during the study period. The dotted line shows the trend of moving averages of the number of survivors.

Bacterial pneumonia is a frequent etiology of acute respiratory failure requiring ECMO. The mortality in this series was similar to that reported in adults.^(^17^)^ We observed an increase in survival rates in cases of ECMO use due to this condition, from 60.4% to 66.1%, although it did not reach statistical significance. As the number of runs analyzed was low, we suspect that this improvement could be significant with an increase in the number of cases evaluated.

Our study is based on a registry that does not collect information regarding long-term outcomes such as disabilities and quality of life. Therefore, the real impact of ECMO cannot be inferred, and this is an important limitation. We also lack information on other conditions that affect mortality, such as nonpulmonary organ failure, the presence of chronic pulmonary diseases at the time of ECMO treatment, or even demographic data, such as age. The data were self-reported by each institution and not validated by other investigators. Variations in practices may have influenced the results as well, and the clinical database does not include a severity of illness score. However, given the paucity of studies in pediatrics, the ELSO registry has been used to help answer many research questions. The observed improvement in survival detected in the current study can be important in aiding clinicians in patient selection for ECMO support and in counseling families regarding prognosis.

## CONCLUSION

The 2009 H1N1 outbreak provided an opportunity for several centers to use extracorporeal membrane oxygenation as a rescue therapy for severe acute respiratory failure in children. In subsequent years, there was a significant increase in the survival rates among children receiving extracorporeal membrane oxygenation for acute respiratory failure related to viral pneumonia and other respiratory illnesses.
